# Honoring Choice in Grief Through Expressive Arts With Long-Term Care Residents

**DOI:** 10.1093/geroni/igaa057.2348

**Published:** 2020-12-16

**Authors:** Michelle Olson

**Affiliations:** Concordia University Chicago, Fishkill, New York, United States

## Abstract

Death in long-term residential care homes is a common occurrence, yet it is often taboo and strongly avoided. Staff and residents often express deep connections to one another in these settings, but when death occurs, there is often little to no support, training or space to share these feelings. This session will discuss the findings of Dr. Olson’s multicase, arts-based research from the elder voices of those who face these losses. Perceptions such as disenfranchised grief and ageism were revealed in this study as well as positive expressions such as love, kindness and acceptance. The shared findings will include poetry and artwork that was created within this research study. Utilizing the creative arts can assist in the expression of these complex and abstract human emotions, instill a sense of comfort and community and empower honor these lives and friendships.

